# Making predictions under interventions: a case study from the PREDICT-CVD cohort in New Zealand primary care

**DOI:** 10.3389/fepid.2024.1326306

**Published:** 2024-04-03

**Authors:** Lijing Lin, Katrina Poppe, Angela Wood, Glen P. Martin, Niels Peek, Matthew Sperrin

**Affiliations:** ^1^ Division of Informatics, Imaging and Data Science, Faculty of Biology, Medicine and Health, University of Manchester, Manchester, United Kingdom; ^2^ Schools of Population Health & Medicine, Faculty of Medical and Health Sciences, University of Auckland, Auckland, New Zealand; ^3^ British Heart Foundation Cardiovascular Epidemiology Unit, Department of Public Health and Primary Care, University of Cambridge, Cambridge, United Kingdom; ^4^ British Heart Foundation Centre of Research Excellence, University of Cambridge, Cambridge, United Kingdom; ^5^ National Institute for Health and Care Research Blood and Transplant Research Unit in Donor Health and Behaviour, University of Cambridge, Cambridge, United Kingdom; ^6^ Health Data Research UK Cambridge, Wellcome Genome Campus and University of Cambridge, Cambridge, United Kingdom; ^7^ Cambridge Centre of Artificial Intelligence in Medicine, University of Cambridge, Cambridge, United Kingdom

**Keywords:** clinical prediction model, causal inference, cardiovascular diseases, prevention, treatment

## Abstract

**Background:**

Most existing clinical prediction models do not allow predictions under interventions. Such predictions allow predicted risk under different proposed strategies to be compared and are therefore useful to support clinical decision making. We aimed to compare methodological approaches for predicting individual level cardiovascular risk under three interventions: smoking cessation, reducing blood pressure, and reducing cholesterol.

**Methods:**

We used data from the PREDICT prospective cohort study in New Zealand to calculate cardiovascular risk in a primary care setting. We compared three strategies to estimate absolute risk under intervention: (a) conditioning on hypothetical interventions in non-causal models; (b) combining existing prediction models with causal effects estimated using observational causal inference methods; and (c) combining existing prediction models with causal effects reported in published literature.

**Results:**

The median absolute cardiovascular risk among smokers was 3.9%; our approaches predicted that smoking cessation reduced this to a median between a non-causal estimate of 2.5% and a causal estimate of 2.8%, depending on estimation methods. For reducing blood pressure, the proposed approaches estimated a reduction of absolute risk from a median of 4.9% to a median between 3.2% and 4.5% (both derived from causal estimation). Reducing cholesterol was estimated to reduce median absolute risk from 3.1% to between 2.2% (non-causal estimate) and 2.8% (causal estimate).

**Conclusions:**

Estimated absolute risk reductions based on non-causal methods were different to those based on causal methods, and there was substantial variation in estimates within the causal methods. Researchers wishing to estimate risk under intervention should be explicit about their causal modelling assumptions and conduct sensitivity analysis by considering a range of possible approaches.

## 1 Background

Prognostic clinical prediction models (CPMs) provide assessments of individual risk of future adverse outcomes, conditional on characteristics available at the time that the prediction is made (1). CPMs have a wide range of uses; among these is to provide clinical decision support. For example, QRISK is a CPM for cardiovascular disease, used in UK primary care, to determine whether it is appropriate to offer a patient a statin (2), according to whether a patient exceeds an absolute risk of 10% within a 10-year timeframe. Decision rules such as this are commonly used, based on the common finding that benefits from intervention are higher in patients with higher absolute risk, while harms (or costs) are fixed or increase more slowly (3).

CPMs are used to guide intervention choices (4), but do not tell us how those interventions will affect the individual's risks of future adverse outcomes. As noted by Hernán et al. (5) “predictive algorithms inform us that decisions have to be made, but they cannot help us make the decisions.”

One way in which this might be done, as observed in practice (6), is by modifying the inputs to the predictive algorithm from the actual characteristics of the patient to the expected characteristics after an intervention—which we call here *conditioning on interventions in non-causal models*. We believe this approach is widespread in healthcare practice (7). However, this approach is not expected to provide an accurate estimate of the change in predicted risk as a result of a planned intervention (8); to do so requires causal inference techniques (9, 10).

CPMs are rarely developed with explicit consideration of prediction under intervention [with notable exceptions (8, 11)]. However, methods are emerging to allow for this (12). In a recent scoping review on methods enabling prediction under intervention, two main classes of approach were identified: to use externally estimated causal effects (e.g., from clinical trials) combined with a standard prediction model, or to develop a CPM that allows for prediction under intervention by combining CPM development techniques with causal inference methods for observational data.

To the best of our knowledge, no studies have empirically compared different methods for prediction under intervention. Therefore, we aimed to demonstrate and compare methods, both from the two main classes of approach, and the approach of conditioning on interventions in non-causal models (since this is an approach likely used in practice), in terms of their estimated absolute risks.

## 2 Methods

### 2.1 Data source

PREDICT is a prospective open cohort study set in New Zealand (13). Participants are automatically enrolled when primary healthcare practitioners complete standardised cardiovascular disease (CVD) risk assessments using a web-based CVD risk assessment and management decision support system. New Zealand CVD risk management guidelines recommend such CVD risk assessments begin on men aged 45 years and women aged 55 years (or 10 years earlier for people of higher CVD risk ethnicities), with the frequency of subsequent assessment and intensity of risk management informed by their calculated 5-year CVD risk. Participant risk factor profiles captured by the software are regularly linked to national databases documenting drug dispensing and ICD-coded hospitalisations and deaths related to cardiovascular diseases. The cohort is representative of the source population and the PREDICT system is now being used in about one third of New Zealand's population. Data is complete on the mandatory variables required for CVD risk assessment: age, sex, ethnicity, previous diagnosis of CVD, diabetes, atrial fibrillation, a self-reported family history of premature ischaemic CVD, smoking status, systolic and diastolic blood pressure (SBP, mean of two measures) and total cholesterol to high-density lipoprotein-cholesterol ratio (TC/HDL-C ratio, one measure). Other lipid fractions, body mass index (BMI) and dispensed cardiovascular medications [classified into blood pressure (BP)-lowering and lipid-lowering medications, antiplatelet and anticoagulant agents] may also be filled in but are not compulsory for CVD risk assessment. These variables are routinely entered if clinicians require individualised guideline-based recommendations for patient management. Dispensed cardiovascular medications, hospitalizations, deaths, along with New Zealand Index of Socioeconomic Deprivation (i.e., NZDep scores) can also be obtained from the linked national databases. For the current study, we used data collected between 20 October 2004 and 11 October 2018.

We defined the primary outcome to be CVD-related hospital admission or death, in line with existing PREDICT models (14) predicting 5-year incident CVD risk. Time on study was the time from each patient's first (baseline) PREDICT assessment to the first of the following: hospital admission or death related to CVD, death from other causes, or end of follow-up (31 December 2018). The cohort has almost complete ascertainment of CVD events, as more than 95% of patients with an acute CVD event in New Zealand are managed by public health services (13), and therefore we assume that all patient outcomes before the cohort cut-off time are known.

### 2.2 Overview of statistical approach

We focus on the pragmatic approach of augmenting a standard prediction model with the ability to predict under intervention, as opposed to developing an entirely new model with causal consideration from the beginning.

The PREDICT model (14) was developed using Cox proportional hazards modelling with all pre-specified variables in Table 1. Using standard prediction modelling techniques, we first refitted the PREDICT model to our study cohort as the cohort was updated compared with that used to fit the published PREDICT model. This was done by re-calculating the coefficients and the baseline hazard while keeping the same structure of the PREDICT equations. Models are separate for males and females. Note in the PREDICT model, interaction terms, such as those involving blood pressure and medication, were included if they met a strict predetermined threshold statistical significance of *p* < 0.001 and were clinically plausible and if the plotted data suggested effect modification. For more details on the development of the PREDICT model, refer to (14).

**Table 1:** Baseline characteristics and outcomes of the PREDICT cohort.

	Total	With follow-up within 2 years from baseline	No follow-up within 2 years from baseline
	(*N* = 453,451)	(*N* = 110,250)	(*N* = 343,201)
Age(years), mean (SD)	53 (9.8)	55 (10)	53 (9.6)
Sex			
Female	199,256 (44%)	47,752 (43%)	151,504 (44%)
Male	254,195 (56%)	62,498 (57%)	191,697 (56%)
SBP, Mean mmHg (SD)	130 (15)	130 (16)	130 (15)
TC/HDL, mean (SD)	4.1 (1.2)	4.3 (1.3)	4.0 (1.2)
Incident total CVD within 5 yearrs from baseline	15,577 (3%)	4,531 (4%)	11,046 (3%)
Ethnicity			
European	244,543 (54%)	55,077 (50%)	189,466 (55%)
Others	208,908 (46%)	55,173 (50%)	153,735 (45%)
NZ Dep, mean (SD)	3.0 (1.5)	3.1 (1.5)	2.9 (1.5)
Smoking			
Current smoker	66,458 (15%)	18,258 (17%)	48,200 (14%)
Ex-smoker	76,539 (17%)	20,779 (19%)	55,760 (16%)
Never smoker	310,454 (68%)	71,213 (65%)	239,241 (70%)
Family history of premature CVD	46,819 (10%)	14,179 (13%)	32,640 (10%)
Diabetes	48,992 (11%)	31,589 (29%)	17,403 (5%)
Atrial fibrillation	6,107 (1%)	1,808 (2%)	4,299 (1%)
Medications at baseline			
Lipid-lowering medication	75,485 (17%)	32,023 (29%)	43,462 (13%)
Antithrombotic medication	45,479 (10%)	20,446 (19%)	25,033 (7%)
BP-lowering medication	105,705(23%)	41,342(37%)	64,363(19%)

The table describes both the complete cohort (*N* = 453,451) as well as the groups that were included (*N* = 110,250) and excluded (*N* = 343,201) in the IPW approach, where we included only people with at least 2 years of follow-up. Data are N (%) unless indicated otherwise. NZ Dep = New Zealand Index of Socioeconomic Deprivation (a numeric value from 1 to 5, lowest to highest deprivation quintile). SBP = systolic blood pressure. TC/HDL = total cholesterol to HDL cholesterol ratio.

For each relevant individual, we wish to estimate the absolute CVD risk with and without interventions for the following scenarios. In each case, we are interested in the situation where the intervention is made at or shortly after an individual has their baseline CVD risk assessment. (I) Smoking cessation: a current smoker stops smoking, (IIa) Reducing blood pressure: a patient with high blood pressure (> = 140 mmHg systolic, stage 2 hypertension) reduces their blood pressure to 130 mm Hg or below through means other than medication (such as lifestyle modification), (IIb) Blood pressure lowering medication (BPLM) initiation: a patient with untreated hypertension initiates BPLM, (IIIa) Reducing cholesterol: a patient with high cholesterol (TC/HDL-C ratio > = 5) reduces their cholesterol ratio to 3.5 or below through means other than medication (such as lifestyle modification), and (IIIb) Lipid lowering medication (LLM) initiation: patient with high cholesterol initiates LLM.

We compared three main modelling strategies. (a) Conditioning on interventions in non-causal models, in which the intervention was simulated by changing the relevant predictor variable(s) as an input to the CPM. (b) A CPM with intervention effects estimated separately (but using the same data) via inverse probability weighting (IPW). Finally, (c) combining a CPM with causal effects reported in published studies. For approaches (b) and (c), once the intervention effect is estimated or obtained from external studies, for example in the form of relative risk ($RR_{\mathrm{int}}$), risk under intervention can be obtained as $\mathrm{Ris}k_{\mathrm{int}}=\;\mathrm{Ris}k_{\mathrm{original}}\cdot \;RR_{\mathrm{int}}$. Therefore, these approaches assume that treatment effects remain constant on the relative scale. We assess the three approaches by comparing their estimated relative risk (RR) and absolute risk under intervention within both the overall target population and specific population subgroups, including distinctions based on gender (male vs. female), age (<= 50 vs. >50), and ethnicity (European vs. non-European).

We now describe each of these approaches in detail. Details regarding intervention definition, target population, and target estimand are summarised in Table 2.

**Table 2:** Target population, estimand and intervention definition for different approaches in five scenarios: smoking cessation, reducing BP through means other than medication (such as lifestyle), BP lowering medication, lowering cholesterol through lifestyle, and lipid-lowering medications.

Scenarios	Target population for CVD prevention	Target estimand	Non-causal approach to estimate absolute risk under intervention	Causal approach (IPW) Treatment groups for patients in the target population at baseline:
(I) Smoking cessation	All current smokers at baseline	Effect of current smoker quitting smoking now.	Change the value of “smoking” from current smoker to ex-smoker while keeping the remaining risk factors the same	A=1 “ex-smoker” observed at follow-up within two years of the baseline^b^; A=0 “current smokers” observed at follow-up
(IIa) Lowering SBP	All patients with hypertension (SBP> = 140) at baseline	Effect of patients with hypertension reducing BP to normal (< = 130).	Change the value of SBP from whatever its current value is to 130 while keeping the remaining risk factors the same.	A=1: SBP reduction to <= 130 at follow-up within two years of the baseline^b^; A=0: otherwise
(IIb) Blood pressure lowering medication	All patients with untreated hypertension (i.e., SBP> = 140 & BPLM = 0) at baseline	Effect of patients with untreated hypertension initiating BP lowering medications	Change the value of BPLM from 0 to 1 and the value of SBP to 130 while keeping the remaining risk factors the same.^a^	A=1: BPLM =1 at follow-up within two years of the baseline^b^; A=0: otherwise
(IIIa) Lowering TC/HDL-C	All patients with high cholesterol (TC/HDL-C> = 5) at baseline	Effect of patients with high cholesterol reducing TC/HDL-C to normal.	Change the value of TC/HDL-C from whatever its current value is to 3.5 while keeping the remaining risk factors the same.	A=1 TC/HDL-C reduction to <= 3.5 at follow-up within two years of the baseline^b^; A=0: otherwise
(IIIb) Lipid lowering medication	All patients with untreated high cholesterol (TC/HDL-C> = 5 & LLM = 0) at baseline	Effect of patients with untreated high cholesterol initiating lipid lowering medications	Change the value of LLM from 0 to 1 and the value of TC/HDL-C to 3.5 while keeping the remaining risk factors the same.^a^	A=1 LLM =1 at follow-up within two years of the baseline^b^; A=0: otherwise

^a^ Alternatively, one can calculate the risk from keeping the value of SBP (or TC/HDL-C) the same and only changing the value of medication.

^b^ Records from the latest follow-up visit within two years of the baseline were used. The same applied for IPW approach in all five scenarios.

### 2.3 Approach (a): conditioning on interventions in non-causal models

In this approach, hereafter the “non-causal approach”, we represented the intervention risk for an individual by modifying the relevant baseline covariate(s) in the refitted PREDICT model. Specifically, the refitted PREDICT model was used with adjustments made to all relevant baseline covariates for a given intervention, for all relevant patients. For example, for smoking cessation scenario (I), given a risk factor profile of a current smoker, we used the refitted PREDICT model to get an absolute risk prediction if no action was taken. We then changed the value of “smoking” from *current smoker* to *ex-smoker* while keeping the remaining risk factors the same to obtain an absolute risk prediction under quitting smoking. For blood pressure lowering medication scenario (IIb), we changed the value of BPLM from 0 to 1 and the value of SBP to 130 while keeping the remaining risk factors the same. Alternatively, one can also calculate the intervention risk by keeping the value of SBP and only changing the value of medication. This also applied for lipid-lowering medication scenario (IIIb); for these two scenarios, we include results from both options in this study for comparison. Table 2 details how intervention risks were calculated for all scenarios.

This approach readily provides absolute risks and absolute risk reductions. To estimate the overall relative risks, we first computed the individual relative risk as the ratio of the intervention risk with the modified baseline covariate(s), divided by the individual's absolute risk prediction under no action. We then averaged them to obtain the overall relative risk. We emphasise that this approach is not based on causal theory; it assumes that the model coefficient coincides with the causal effect, which will only be the case under very restrictive assumptions, including that the other variables included in the model form a valid adjustment set (6). It has been found to have reasonable performance in specific scenarios (6), but there are no general guarantees. It is included as it is likely used widely in practice.

### 2.4 Approach (b): models with treatment effect estimated via inverse probability weighting (IPW)

We propose a 2-stage method where the absolute risk without intervention, and relative causal treatment effect, are estimated separately. As in the non-causal method, we took the actual risk from the refitted PREDICT model as “*absolute risk without intervention*”. We then combined this risk with a single average treatment effect estimated from the same data using inverse probability weighting (IPW), as explained below, to obtain “*absolute risk under intervention*”. In contrast to methods like stratification using estimated propensity scores for estimating causal effects, IPW-based methods demonstrate consistency with sample sizes and offer approximately unbiased inference for practical sample sizes, and are therefore recommended for routine use in practical applications (15). This approach requires correct specification of the causal structure of the problem, and three key assumptions: exchangeability, consistency, and positivity (16).

For estimating causal effects using IPW, the initial step involves defining the treatment groups (both treated and untreated), followed by identifying potential confounders to adjust for. In our approach, the statuses of being treated and untreated are determined based on both baseline and follow-up data. This approach guarantees that the intervention time closely follows the risk assessment. In the PREDICT cohort, the follow-up period was aligned with routine clinical practices. Repeated assessments of risk factors were performed and documented when considered relevant by either the patient or the practitioner. For each patient, we utilised clinical data from the two-year window following their initial assessment visit. If multiple visits occurred during this time frame, the data from the one closest to the 2-year mark were used. If no follow-up visits took place, the patient was excluded from the analysis. Consequently, the index date was established as the latest follow-up visit within two years of the baseline.

To address potential selection bias within the included group, we calculated the probability of patients returning for visits within the two-year timeframe. These probabilities were incorporated as weights for the subsequent IPW analysis. Further information about the variables used to determine the weight for each selected individual is in the Supplementary Materials. Since the choice of various cut-off times may result in over/under-estimated effects, as a sensitivity analysis, we also present findings from analyses that utilised data from the first follow-up for effect estimation.

For example, when examining smoking cessation, our focus was on current smokers at baseline. Here, we designated A=1 if we observed the status “ex-smoker” at the follow-up visit, indicating successful cessation. Conversely, we assigned A=0 if individuals remained “current smokers” at follow-up. Detailed definitions for all treatments are outlined in Table 2.

For each intervention, after defining the treatment group, we identified confounding factors using a practical variable selection approach based on background knowledge when the causal structure was only partially known, as recommended in (17). We adjusted for all pre-treatment covariates that could be risk factors for the outcome (18). Known causal structures were used to exclude strong instruments and colliders, as these could otherwise introduce biases (19, 20). See the Supplementary Material for detailed confounder identification methods, with relevant confounder variables listed in Table 3.

**Table 3:** Variables used in the treatment model logitPr(A=1|L) and the censoring model $logit\mathrm{Pr}(C_{k}=0|C_{k-1}=0,\;A,\;L,X_{0})$ (see supplementary materials) in the IPW approach for each case.

Treatment A	IPW approach: use post-baseline data to obtain sample treatment status	
	$X_{0}$	L
(I) Smoking cessation	• Baseline diabetes status, atrial fibrillation status, SBP, TC/HDL-C, BPLM, ATM (anti thrombotic medication), LLM	• Sex, age, ethnicity, socioeconomic status, family history of CVD • Baseline diabetes status, atrial fibrillation status • Baseline SBP, TC/HDL-C, ATM, LLM, BPLM
(IIa) Lowering SBP	• Baseline diabetes status, atrial fibrillation status, ATM, LLM	• Sex, age, ethnicity, socioeconomic status, and family history of CVD • Baseline smoking status • Baseline diabetes, atrial fibrillation • Baseline SBP, TC/HDL-C, ATM, LLM, BPLM
(IIb) BP lowering medication	• Baseline diabetes status, atrial fibrillation status, ATM, LLM	• Sex, age, ethnicity, socioeconomic status, and family history of CVD • Baseline smoking status • Baseline diabetes, atrial fibrillation • Baseline SBP, TC/HDL, ATM, LLM
(IIIa) Lowering TC/HDL-C	• Baseline diabetes status, atrial fibrillation status, ATM, BPLM	• Sex, age, ethnicity, socioeconomic status, and family history of CVD • Baseline smoking status • Baseline diabetes, atrial fibrillation • Baseline SBP, TC/HDL-C, ATM, LLM, BPLM
(IIIb) Lipid lowering medication	• Baseline diabetes status, atrial fibrillation, BPLM, ATM	• Sex, age, ethnicity, socioeconomic status, and family history of CVD • Baseline smoking status • Baseline diabetes, atrial fibrillation • Baseline SBP, TC/HDL, ATM, BPLM

Once the treatment and confounders were established, the next step involved specifying models for estimating inverse probability weights and effects. Given that the confounding factors for different treatments varied (21), we constructed models for each intervention (I-III) separately. Let L represent the set of identified confounding factors for intervention A, and let $X_{0}$ denote the other prognostic factors for the outcome (Figure E1). We applied standard IPW methods (16) with stabilised treatment weights, W(A)=Pr(A)/Pr(A|L), calculated via the treatment probability Pr(A=1) and the conditional probability Pr(A=1|L) given the variables in L. These probabilities are calculated by fitting the following logistic models.

$$\begin{array}{c} \mathrm{logit}\mathrm{Pr}(A=1)=\gamma_{0},\;\\ \mathrm{logit}\mathrm{Pr}(A=1|L)=\alpha_{0}+\alpha_{1}l_{1}+\alpha_{2}l_{2}+\cdots +\alpha_{d}l_{d}. \end{array}$$

To minimise the risk of model misspecification, we compare linear models with more flexible cubic spline specifications and asses the models using the Akaike Information Criterion (AIC). The estimated stabilised IP weight is then Pr(A=1)/Pr(A=1|L) for the treated and (1−Pr(A=1))/(1−Pr(A=1|L)) for the untreated. Further details and advantages of using stabilised weights can be found in (22, 23). Selection bias due to administrative cut-off and loss to follow-up (including competing events) was adjusted for by conceptualising censoring as a time-varying treatment and applying standard IPW for time-varying treatments (22, 23). With the calculated weights for both treatment and censoring, we then estimated treatment effect in a hazard model for the outcome. Briefly, we applied an inverse probability weighted pooled logistic model to estimate the following parameters.

$$\begin{array}{c} \;\mathrm{logit}\mathrm{Pr}(D_{k+1}^{\alpha }=1|D_{k}^{\alpha }=0)=\beta_{0}+\beta_{1}k+\beta_{2}\alpha +\;\beta_{3}\alpha \times k, \end{array}$$

where $D_{k}$ indicate CVD status at time k (months of follow-up). Then the probability of survival that would have been observed, i.e., $\mathrm{Pr}(D_{k+1}^{\alpha }=0)$, with α=1 for under treatment or α=0 for no treatment, can be obtained by multiplying $1-\mathrm{Pr}(D_{k+1}^{\alpha }=1|D_{k}^{\alpha }=0)$ over the time k. Further details of the weight and effect models can be found in the Supplementary Materials.

### 2.5 Approach (c): combining with causal effects measured externally

This approach is similar to approach (b) in that risks without intervention were estimated from the refitted PREDICT model, and combined with relative effect estimates of interventions for computing risk under intervention. In this case, relative effect estimates were obtained from the literature rather than the data in hand. The approach assumes that the estimated effect has external validity in the population of interest. We used the same effect estimates as the Million Hearts tool [Table 3 (8)], which was informed by an overview of systematic reviews (24). The overview selected a total of 35 systematic reviews investigating the effects of several interventions in primary prevention of atherosclerotic cardiovascular. Specifically, the estimated overall CVD risk reduction, expressed as relative risk (RR), for smoking cessation was 0.73. Blood pressure-lowering therapy resulted in an overall CVD risk reduction of 0.73, and the RR for lowering SBP (by changing lifestyle) was 0.65. Both the use of lipid-lowering medication (statins) and reducing lipid levels through lifestyle changes yielded a 25% decrease in major CVD events.

All the methods and comparisons in this study were implemented in R version 4.1.1, and the code is available on GitHub (https://github.com/manchester-predictive-healthcare-group/predictions-under-intervention).

## 3 Results

### 3.1 Baseline information

The study population comprised 453,451 participants aged 30–74 years at the time of their recruitment to the study between 20 Oct, 2004, and 11 Oct, 2018*.* The baseline characteristics for the cohort are provided in Table 1. Kaplan-Meier plots of survival probabilities in different patient subgroups are in the Supplementary Figure E2. As noted in Methods, there was no missing data for these mandatory variables required for the CVD risk assessment using PREDICT software. For estimating average treatment effects using the IPW, only patients followed up within 2 years were used. Table 1 reports the baseline characteristics for the excluded vs. included patients; there were significant differences between the two groups. The selection bias was corrected by IP weighting as described in Methods. Additionally, among individuals with follow-up data, 2019 of the 18,258 baseline current smokers quit smoking. In other words, 11% transitioned from being current smokers to ex-smokers between baseline and follow-up. The average changes in SBP and TC/HDL-C among these individuals were a reduction of 1.06 mmHg and 0.08, respectively, between baseline and follow-up (Supplementary Table E1).

### 3.2 Refit the PREDICT CPM

The original PREDICT model (14) predicting incident CVD risk was developed using Cox regression models including risk factors listed in Table 1. The regression coefficients of the original PREDICT model and of the refitted PREDICT model from the cohort used in this study are compared in the Supplementary Table E2. The largest relative change in regression coefficients between two models was in the coefficient for Pacific ethnic group. The predicted 5-year CVD risks in the current study cohort from refitted PREDICT model were 4.28% for men and 3.01% for women on average.

### 3.3 Causal effects reported in the literature

The effects of interventions that correspond to our different scenarios, as derived from (8) are summarised in Table 4. The relative risk for smoking cessation was 0.73 (95% CI, 0.62–0.85). The original paper lacked a 95% confidence interval; we derived an estimate using the provided data.

**Table 4:** Relative risks (RR) for interventions estimated by the different methods: means and 95% confidence intervals (CI).

	Non-causal approach using refitted PREDICT, mean RR (95% CI)	IPW approach RR (95% CI)	Effects from other sources RR (95% CI)
(I) Smoking cessation	0.63 (0.56–0.70)	0.70 (0.20–1.20)	0.73 (0.62–0.85)^c^
(IIa) Lowering SBP	0.70 (0.47–0.94)	0.93 (0.54–1.31)	0.65 (0.57–0.75)^d^
(IIb) BP lowering medication	1.12 (0.85–1.40)^a^	1.16 (0.69–1.62)	0.73 (0.67–0.81)
(IIIa) Lowering TC/HDL-C	0.73 (0.58–0.88)	0.91 (0.24–1.53)	0.75 (0.70–0.80)^e^
(IIIb) Lipid lowering medication	0.71 (0.57–0.86)^b^	1.02 (0.43–1.58)	0.75 (0.70–0.81)

^a^ Effect estimated for patients taking medication and reducing SBP to reduce to 130; if the SBP reduction was not imposed, the RR was 1.41 (1.30–1.52).

^b^ Effect estimated for patients taking medication and reducing TC/HDL-C to reduce to 3.5; if the TC/HDL-C reduction was not imposed, the RR was 0.97 (0.92–1.03).

^c^ The original paper did not include a 95% CI; we calculated an estimate using the provided data.

^d^ For lowering blood pressure, external effects are RR for patients reducing per 10 mm Hg after taking medication.

^e^ For lowering cholesterol, external effects are treatment effects standardized per 1 mmol/L reduction in levels of low-density lipoprotein cholesterol.

Blood pressure-lowering therapy reduced overall CVD risk by a RRR (relative risk reduction) of 0.73 (95% CI, 0.67–0.81), and a RRR of 0.65 (95% CI, 0.57–0.75) was observed per 10 mmHg actual SBP lowering. We therefore used the RRR of 0.65 for blood pressure lowering scenario (IIa) and RRR of 0.73 for blood pressure medication scenario (IIb) in this study.

The use of lipid lowering medication (statins) led to a 25% reduction in major CVD events (RRR 0.75; 95% CI, 0.70–0.81); a 1 mmol/L reduction in LDL-cholesterol had a RRR of 0.75 (95% CI, 0.70–0.80). Of note, we did not find papers estimating direct effects of TC/HDL-C ratio reduction on CVD risk. We therefore used the RRR of 0.75 for lipid-lowering medications scenario (IIIb), and the effect of per 1 mmol/L reduction in LDL-cholesterol on CVD (RRR 0.75) for cholesterol lowering scenario (IIIa), given the lack of direct evidence available on this scenario.

### 3.4 Relative risk estimates

Table 4 reports the relative effects of intervention (in terms of relative risk of the treated v*s.* untreated) estimated from the compared approaches. All approaches generated similar estimates for the effects of smoking cessation on CVD risk. For the effects of lowering SBP (IIa) and lowering TC/HDL-C (IIIa), the IPW approach produced the most conservative treatment effect estimates among all approaches.

When estimating the effects of initiating blood pressure lowering medication (IIb) or lipid lowering medication (IIIb) on CVD risk, regardless of actual blood pressure or cholesterol reduction within a certain timeframe, estimated treatment effects varied. Estimates from IPW indicated an elevation in an individual's CVD risk upon initiating medication. Notably, for the blood pressure lowering medication intervention, only the approach of incorporating a trial estimated effect led to a reduced risk, whereas all other approaches estimated an increase in risk. The sensitivity analysis results reveal minimal differences in the estimated relative risks using the IPW approach when comparing data from the 1st follow-up after baseline and data from the visit closest to the 2-year mark after baseline (Supplementary Table E3). The Supplementary Figure E3 shows the estimated stabilised IP weights for each intervention. Upon comparing linear treatment models with more flexible cubic spline specifications, the AIC indicated that the additional complexity introduced by the spline terms did not enhance the model fit (Supplementary Table E4). Figure E4 shows the estimated survival curves for untreated vs. treated participants under each IPW hazard model.

### 3.5 Absolute risk under interventions

**Figure 1:**
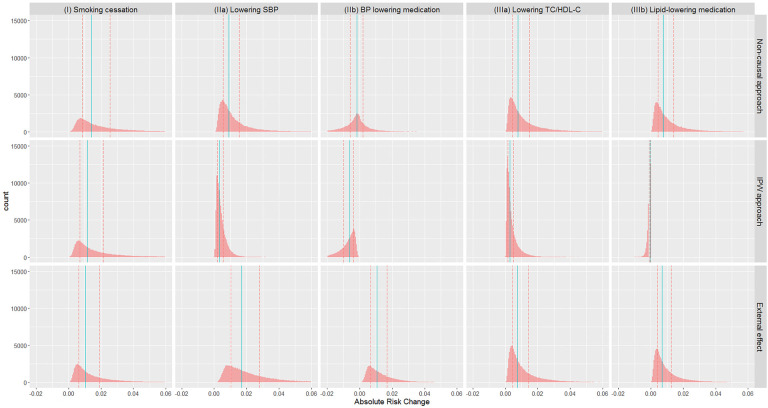
Distribution of absolute risk changes (ARC) estimated from different approaches in different scenarios: (I) smoking cessation, (IIa) lowering SBP, (IIb) BP lowering medication, (IIIa) lowering TC/HDL-C, and (IIIb) lipid lowering medication. ARC = risk without intervention—risk under intervention. Red dashed lines: lower and upper quartiles; blue solid lines: median.

Absolute risks without intervention, and under each intervention based on each approach, are summarised in Table 5 (visualised in the Supplementary Figure E5), while absolute risk change (ARC) distributions are visualised in Figure 1 (ARC = risk without intervention—risk under intervention; numerical results in the Supplementary Table E5). The median absolute risks without intervention varied as the target populations differ. For (I) smoking cessation, absolute risk reduction median varied from 1.0% to 1.4% depending on methodological approach (Figure 1). For (IIa) lowering SBP, absolute risk reductions varied substantially between approaches, while for (IIb) BP lowering medication, absolute risk changes were both positive and negative, and highly variable. Similar findings were observed for the cholesterol scenarios (IIIa) and (IIIb).

**Table 5:** Absolute risk: median and lower and upper quartiles (LQ, UQ) across the target population, estimated from different approaches.

	Current risk (without intervention): median (LQ, UQ)	Absolute risk under hypothetical interventions median (LQ, UQ)		
		Non-causal approach	Causal approach (IPW)	Using external effect estimates
(I) Smoking cessation	3.89% (2.30%, 7.11%)	2.46% (1.45%, 4.54%)	2.73% (1.61%, 4.97%)	2.84% (1.68%, 5.19%)
(IIa) Lowering SBP	4.88% (2.97%, 7.96%)	3.68% (2.15%, 6.28%)	4.54% (2.76%, 7.40%)	3.18% (1.93%, 5.17%)
(IIb) BP lowering medication	3.91% (2.47%, 6.20%)	3.93% (2.46%, 6.42%)	4.53% (2.87%, 7.20%)	2.85% (1.81%, 4.53%)
(IIIa) Lowering TC/HDL-C	3.06% (1.74%, 5.69%)	2.24% (1.28%, 4.15%)	2.78% (1.58%, 5.18%)	2.29% (1.31%, 4.27%)
(IIIb) Lipid lowering medication	2.79% (1.65%, 5.02%)	1.99% (1.18%, 3.58%)	2.85% (1.68%, 5.12%)	2.09% (1.23%, 3.77%)

The median absolute risk changes varied across different patient subgroups, i.e., *Female* vs. *Male*, *Age < = 50* vs. *Age >50*, and *European* vs. *Non-European* (Table 6). For all five scenarios, the largest group difference in absolute risk change estimated from the IPW approach was observed in *Age < = 50* vs. *Age >50* groups.

**Table 6:** Absolute risk changes (ARC) in different subpopulations: median and lower and upper quartiles (LQ, UQ). ARC = risk without intervention—risk under intervention.

	Sex		Age		Ethnicity	
	Female	Male	Age < = 50	Age >50	European	Non-European
(A) ARC from the non-causal approach						
(I) Smoking cessation	1.41% (0.85%, 2.511%)	1.43% (0.84%, 2.59%)	1.00% (0.66%, 1.50%)	2.39% (1.53%, 3.79%)	1.53% (0.94%, 2.60%)	1.35% (0.79%, 2.54%)
(IIa) Lowering SBP	0.68% (0.41%, 1.18%)	1.09% (0.70%, 1.84%)	0.90% (0.55%, 1.59%)	0.90% (0.54%, 1.54%)	0.80% (0.51%, 1.31%)	1.08% (0.62%, 1.95%)
(IIb) BP lowering medication	−0.25% (−0.62%, 0.043%)	−0.12% (−0.58%, 0.30%)	0.005% (−0.14%, 0.44%)	−0.39% (−0.87%, −0.09%)	−0.25% (−0.67%, 0.09%)	−0.08% (−0.45%, 0.33%)
(IIIa) Lowering TC/HDL-C	0.71% (0.38%, 1.33%)	0.81% (0.44%, 1.55%)	0.55% (0.32%, 0.90%)	1.29% (0.77%, 2.22%)	0.84% (0.49%, 1.52%)	0.72% (0.38%, 1.47%)
(IIIb) Lipid lowering medication	0.75% (0.41%, 1.38%)	0.77% (0.44%, 1.43%)	0.55% (0.33%, 0.88%)	1.30% (0.80%, 2.15%)	0.84% (0.50%, 1.49%)	0.70% (0.38%, 1.35%)
	Sex		Age		Ethnicity	
	Female	Male	Age < = 50	Age >50	European	Non-European
(B) ARC from the IPW approach.						
(I) Smoking cessation	1.06% (0.64%, 1.90%)	1.24% (0.72%, 2.27%)	0.80% (0.54%, 1.25%)	1.98% (1.23%, 3.19%)	1.25% (0.77%, 2.17%)	1.11% (0.64%, 2.11%)
(IIa) Lowering SBP	0.28% (0.17%, 0.44%)	0.41% (0.25%, 0.65%)	0.22% (0.15%, 0.34%)	0.41% (0.26%, 0.63%)	0.33% (0.21%, 0.52%)	0.36% (0.21%, 0.62%)
(IIb) BP lowering medication	−0.49% (−0.76%−0.33%)	−0.74% (−1.16%, −0.47%)	−0.43%(−0.63%, −0.31%)	−0.76% (−1.16%−0.49%)	−0.62% (−0.96%, −0.40%)	−0.63% (−1.05%, −0.39%)
(IIIa) Lowering TC/HDL-C	0.26% (0.14%0.48%)	0.28% (0.16%, 0.53%)	0.18% (0.12%, 0.29%)	0.47% (0.29%, 0.78%)	0.30% (0.18%, 0.52%)	0.25% (0.14%, 0.50%)
(IIIb) Lipid lowering medication	−0.05% (−0.09%, −0.03%)	−0.06% (−0.10%, −0.03%)	−0.04% (−0.06%, −0.03%)	−0.10% (−0.16%, −0.06%)	−0.06% (−0.11%−0.04%)	−0.05% (−0.09%, −0.03%)

## 4 Discussion

### 4.1 Main findings

Existing CPMs can inform us that decisions have to be made, but the support they can offer in making those decisions is limited because they do not allow predictions under interventions (5). We have compared different methodological approaches for predicting individual level cardiovascular risk under a range of interventions: smoking cessation, reducing blood pressure, reducing cholesterol, blood pressure lowering medication initiation, and lipid-lowering medication initiation. Our work makes two key contributions to the literature.

First, we illustrate and provide code for a range of “causal prediction” methods (9)—including conditioning on intervention in a non-causal model, estimating causal effects from the data using inverse weighting and combining these causal effects with a prediction model, and combining externally estimated effects with a prediction model. The approach of conditioning on intervention in a non-causal model is simple in that it readily provides risk under interventions for each individual using existing clinical prediction models; however, this approach is not grounded in any causal theory so highly vulnerable to bias when targeting *any* causal estimand. Causal estimation using inverse probability weighting assumes all variables needed to adjust for confounding are identified and correctly measured; the validity of the proposed procedure requires correct specification of both the treatment model and the marginal hazards model. The approach combining externally measured effects is a simple way to combine causal effects with an existing clinical prediction model; however, transportability of the trial effect needs to be assumed.

Second, we illustrate that each of these approaches can give different results, sometimes in contrasting directions, depending on the interventions under consideration. For example, all the approaches estimated similar effects for smoking cessation. The small difference between the non-causal and IPW methods could be attributed to a substantial overlap in the variables already considered in prediction and those used as confounders. On the other hand, for the blood pressure lowering medication intervention, only the approach of incorporating a trial estimated effect led to a reduced risk, with all other approaches estimated an increase in risk, which is implausible so almost certainly reflects residual confounding. Our findings regarding the variations in the estimated effects of different interventions on CVD risk in different subgroups suggest that group-level estimates of changes in risk are likely to be more precise. This precision can be beneficial in clinical practice, aiding in the decision-making process for optimal interventions to those in whom they will provide the best benefit.

### 4.2 Relation with other studies

We are not aware of other studies that have compared different approaches to calculating risk under intervention as we have done here. However, there are studies that used the same, or very similar, approaches described in this paper.

Conditioning on intervention in a non-causal model has a long history, recommended, for example, over 20 years ago by Hingorani and Vallance (6). They acknowledge that the approach is prone to bias, but demonstrate that, for the examples they consider, the results appear to be in line with evidence from randomised controlled trials. This was reflected in our study where we found results for the non-causal approach to often be in line with the causal approaches. Change in risk score is also often used as an endpoint in clinical trials [e.g., (25, 26)], which implicitly assumes that the reduction is an accurate representation of the true risk reduction.

The approach of combining a CPM with externally estimated causal effects was also implemented in the “Million Hearts” study for primary prevention of CVD, and we used their externally estimated causal effects here (8). This approach implicitly assumes that the baseline absolute risk estimate from the CPM represents the “untreated” intervention. This assumption may be violated due to treatment drop-in (27).

A breast cancer model (11) (also called PREDICT but different from the CVD PREDICT model that we focused on) fixed treatment coefficients to estimates from published trials, which is similar to our approach except that we separately estimated treatment coefficients for the prognostic model from those used to estimate risk under intervention. This reflects a slightly different context since the PREDICT breast cancer model is applied at a well-defined time point (immediately after surgery when considering treatment), rather than arbitrary timepoints as is the case with PREDICT-CVD.

The approaches that we have considered in this paper all assume that treatment effects are constant on the relative scale, with heterogeneity on the absolute scale occurring because of variation in baseline absolute risk. This can be extended with individual patient data from randomised trials, for example by fitting separate models in treated and untreated groups to allow full flexibility in treatment contrasts at all covariate levels (28). There are numerous approaches in the machine learning literature addressing this problem—for example, causal forests (29). These approaches can also be applied to observational data under the assumption of conditional exchangeability given all of the included prognostic variables (30).

Consideration of how treatment should be handled when predicting treatment-naïve risk was considered by Groenwold et al. (31), who recommended including a variable for treatment at baseline. This work did not consider comparing absolute risk under different treatments, and indeed did not invoke any causal inference machinery.

Here, we focused on the simpler point treatment case, but similar ideas have also been explored in the context of time-dependent treatment estimation, specifically to address treatment drop-in, where particularly careful consideration of target estimands is required (10). The approach of inverse probability weighting was applied to clinical prediction in Sperrin et al. (27), and similarly, Xu et al. (32) addressed treatment drop-in using externally estimated causal effects.

### 4.3 Strengths and limitations

A strength of the analysis is that we used a real observational dataset that has already been used to develop a prediction model that is used in practice, PREDICT. We also considered a range of interventions—stopping smoking, lowering blood pressure, lowering lipid level, and blood pressure/lipid-lowering medication initiation.

A limitation in terms of generalising these results is that the ground truth is unknown. We cannot assume that externally measured causal effects constitute the ground truth, even if they were obtained from randomised controlled trials—it is not always possible to identify studies evaluating the exact intervention of interest and we cannot assume that results from tightly controlled trials generalise to real-world settings. Nevertheless, we have highlighted the disagreements between different approaches.

Throughout the paper, we have taken the risk from a standard predictive model as absolute risk without intervention. This is not accurate as this is the risk under usual care rather than no intervention; many people in the PREDICT cohort will have received one of the interventions of interest during the follow-up period [so-called treatment drop-in (27)]. A more accurate (but more complex) approach is to directly target risk without intervention using causal considerations (10, 27). This involves estimating a combined model for the prediction and causal parts. We focused here on the more pragmatic approach of augmenting an existing “standard” prediction model with the ability to estimate causal contrasts through a two-stage approach.

We adopted a practical approach to identify confounders for adjustment in the causal model based on causal structure that is only partially known and this is not intended to be definitive. Accurate causal inference would require a robust approach to selecting variables for adjustment with a full causal structure for the domain of interest, including input from experts and a detailed assessment of the literature.

Some of the interventions we propose are non-specific, particularly reducing blood pressure and cholesterol through lifestyle modification. This can lead to violation of the consistency assumption in the causal modelling approaches. Different lifestyle interventions (e.g., targeting diet vs. exercise) are likely to have different effects. However, evidence for lifestyle interventions in general is lacking, and therefore the non-specific approach we have taken may represent the best evidence currently available. We see this as a step forward compared with the non-causal approach, but encourage the use of evidence around more specific interventions where this is available.

We only considered the average treatment effects in the causal models and therefore did not allow treatment effect heterogeneity. However, the IPW procedure can be generalised by including some pre-treatment covariates into the hazard model to allow for effect modification. Conditional average treatment effects can be derived if trial data is available (28). Moreover, approaches are emerging in which trial data and observational data can be formally combined, thereby fully exploiting the complementary strengths of the two sources (33).

A final limitation is that we considered interventions individually and not in combination. It may be that interventions interact with each other to produce larger than expected, or smaller than expected, changes in absolute risk. However, this was studied by Lloyd-Jones et al. (8) who found no strong evidence of interactions.

### 4.4 Implications

The results of this work demonstrate that even with incorporation of causal machinery, considerable thought and care is needed to produce models that can reliably make predictions under interventions, given the inconsistency between approaches.

These inconsistencies are explainable by the different assumptions made by each of the studied methods. We reiterate that the non-causal approach is not grounded in any causal theory, so this approach should be used with extreme caution. It should first be checked that specific input modification leads to results that are in line with expectations given e.g., randomised controlled trial findings (6). As is well established, the inverse probability weighting approach requires a full understanding of the causal structure of the problem, and therefore its use should be accompanied by expert input, and sensitivity analyses to explore the impact of unverifiable assumptions such as conditional exchangeability. The approach of using causal effects estimated elsewhere assumes that the estimated effect has external validity in the population of interest [target validity (34)]. Therefore, trials that are as similar as possible to the target population should be preferred when using this approach, and methods for reweighting populations to overcome a mismatch in measured characteristics should also be used (35).

The methods described make different, complementary, assumptions. While the compatibility of estimates across different methods can be seen as reassuring (16), the inconsistencies between approaches, if any, could provide insight into the validity of the underlying causal assumptions in the models. Therefore, to make clinical predictions under intervention, we suggest that researchers carefully consider the causal knowledge available for the problem, consider all of the methods described here, report the range of potential results, and inspect the resulting effect estimates if incompatible. To facilitate this, we make the code for methods and comparison analyses conducted in this work available.

### 4.5 Unanswered questions and further research

Given the variety of results that this study has found, triangulation of estimated effects from different methodological approaches (both qualitatively and quantitatively) is a key area requiring development and translation to ensure that robust effects are reported, and uncertainty is appropriately represented. Similarly, methods are needed to translate estimated treatment effect heterogeneity on the relative scale to absolute risk, while minimising overfitting and other biases.

An outstanding challenge in this field is validating models that involve making counterfactual predictions. In contrast to “factual” predictions, which can be validated by comparing with observed data, counterfactual models rely on unverifiable assumptions and therefore new approaches need to be identified to validate these models. New methods are emerging for validating counterfactual prediction models, given their critical role in decision-making, particularly in healthcare settings (36–39). In a recent study on validating causal models (37), the authors proposed a qualitative solution that could serve as a causal analogue to the conventional train/test split validation methods used in prediction models. In (38), Boyer et al. examine the conditions under which tailoring a model for counterfactual prediction is possible using training data alone, and further on how to assess the model's performance, and how to perform model and tuning parameter selection. In (39), Keogh and van Geloven, focusing on predictions of time-to-event outcomes, describe how to extend a set of performance measures used in the standard prediction setting to allow for counterfactual performance measurement using artificial censoring and inverse probability weighting.

## 5 Conclusions

Predicting under intervention is clearly desirable when using clinical prediction models for decision support. To do so, it is necessary that the underlying models are carefully constructed based on expert knowledge and using causal inference techniques. Urgent progress is required to increase the robustness of these models given their huge potential impact in decision support scenarios.

## Data Availability

The data analyzed in this study is subject to the following licenses/restrictions: The data that support the findings of this study are available from the University of Auckland PREDICT research steering group, but restrictions apply to the availability of these data, which were used under license for the current study, and so are not publicly available. Requests to access these datasets should be directed to Katrina Poppe, k.poppe@auckland.ac.nz.
