# Supplementary material for: Making predictions under interventions: a case study from the PREDICT-CVD cohort in New Zealand primary care
Source: Front Epidemiol. 2024 Apr 3;4:1326306. doi: 10.3389/fepid.2024.1326306 (PMC11021700; doi:10.3389/fepid.2024.1326306)
Supplement: Supplementary file 1 [file Datasheet1.docx]

Supplementary Material

# Supplementary Methods

## Models eliminating bias in treatment effect via inverse probability weighting (IPW)

The effect estimation follows two steps as detailed in the following. Let $L$ be the set of identified confounding factors and $X_{0}$ be the set of baseline prognostic factors.

1. We estimate the stabilised IP weight for each individual in the study population by fitting logistic models for the probability $Pr(A=1)$ of treatment (e.g., quitting smoking, taking BPLM or LLM) and the conditional probability $Pr(A=1|L)$ given the variables in $L$

$$\begin{aligned} \mathrm{logit}\Pr(A=1)=\gamma_{0} \#(1) \\ \mathrm{logit}\Pr\left( A=1 | L \right)=\alpha_{0}+\alpha_{1}l_{1}+\alpha_{2}l_{2}+\cdots+\alpha_{d}l_{d}.\#(2) \end{aligned}$$

The estimated stabilised IP weight is then $W^{A}= \Pr\left( A=1 \right)/\Pr\left( A=1|L \right)$ for the treated and $(1-Pr \left( A=1 \right))/(1-\Pr\left( A=1|L \right))$ for the untreated.

There exists selection bias due to administrative censoring and loss of follow-up (including competing events). This will be adjusted for by conceptualizing the censoring as a time-varying treatment $C_{1}, \ldots, C_{K}$, where $C_{k}$ is an indicator that takes value 0 if the individual remains uncensored at time $k$ and takes value 1 otherwise; by definition $C_{0}=0$ for all individuals in the study. A pseudo-population if no individuals had been lost to follow-up can be created using the stabilized IP weights

$$\begin{aligned} W^{C}=\prod_{k=1}^{K} \frac{\text{Pr}\left( C_{k}=0 | C_{k-1}=0, A \right)}{\text{Pr}\left( C_{k}=0 | C_{k-1}=0, A,L,X_{0} \right)}, \#\left( 3 \right) \end{aligned}$$

where the numerator and denominator of weights are estimated via two separate pooled logistic models, respectively, as follows

$$\begin{aligned} \mathrm{logit}\Pr\left( C_{k}=0 | C_{k-1}=0, A \right)=\gamma_{0}^{*}+\gamma_{1}^{*}a +\gamma_{2}^{*}k.\#(4) \\ \mathrm{logit}\Pr\left( C_{k}=0 | C_{k-1}=0, A, L,X_{0} \right)=\gamma_{0}++\gamma_{01}a +\gamma_{02}k+ \\ \gamma_{1}l_{1}+\cdots+\gamma_{d}l_{d}+ \gamma_{d+1}x_{1}+\cdots+\gamma_{d+s}x_{s}.\#(5) \end{aligned}$$

Note here we include baseline measurements $X_{0}$ which are associated with both censoring and the outcome in the predictive model for denominator. The effects can then be estimated with IP weighting $W^{A}\times W^{C}$ as in Step 2.

1. We fit a hazards model with individuals that are weighted by their estimated stabilised IP weights using each person-year prior to either a CVD event, censoring, or end of 5-year follow-up (which ever occurred first) as an observation. Let $D_{k}$ indicate CVD status at time $k$ (months of follow-up). This IP weighted pooled logistic model estimates the parameters of the marginal structural logistic model

$$\begin{aligned} \mathrm{logit}\Pr\left( D_{k+1}^{a}=1 | D_{k}^{a}=0 \right)=\beta_{0}+\beta_{1}k+\beta_{2}a+ \beta_{3}a\times k.\#(6) \end{aligned}$$

One minus the estimates of $\Pr\left( D_{k+1}^{a}=1 | D_{k}^{a}=0 \right)$ from the IP weighted hazards model can then be multiplied overtime to obtain an estimate of the survival $\Pr(D_{k+1}^{a}=0)$ that would have been observed under treatment and under no treatment.

## Identify confounders in IPW approaches

There are two general approaches to selecting variables in order to control confounding in estimating causal effects with observational data (1): (a) use of background knowledge and causal graph theory, and (b) empirical approaches that select variables on the basis of statistical associations. A graphical analysis of the structural basis for evaluating confounding is known to be the most robust approach to selecting variables for adjustment; however, construction of a relevant causal structure that is comprehensive and accurate for us to use here is beyond the scope of this paper. We therefore adopt more practical variable selection approaches based on background knowledge when the causal structure is only partially known, as recommended in (1). These strategies include: (a) adjusting for all observed pre-treatment variables that have some connection to the outcome (2), (b) all known risk factors for the outcome (3,4), and (c) all direct causes of the treatment or the outcome (5). We adopted strategy (a) to firstly include for adjustment all pre-treatment covariates that all possible risk factors for the outcome, a strategy often used with propensity score methods. We then used the (partially) know causal structures to exclude, e.g. strong instruments and colliders, as otherwise biases can result (5,6).

## Adjusting for selection bias in the IPW approach

Bias from pre-selecting individuals who followed up within 2 years following their first assessment visit was corrected in the analysis by inverse probability weighting. This was based on assigning a weight $W^{C^{'}}$ to each selected individual ($C^{'}=0$), which was the inverse of the probability of their selection $\Pr{(C}^{'}=0|X^{'})$. We used all mandatory variables required for the CVD risk assessment as variables in $X^{'}$ for estimating the probability, all measured at their first assessment visit: sex, age, ethnicity, socioeconomic status, and family history of CVD, smoking status, diabetes, atrial fibrillation, SBP, TC/HDL-C, ATM, LLM, BPLM. For treatment effect estimation using IPW approach, the IP weighted hazards model was applied using the computed weight $W^{C^{'}}$together with the treatment and censoring weights $W^{A}\times W^{C}$.

# Supplementary Figures and Tables

## Supplementary Figures.


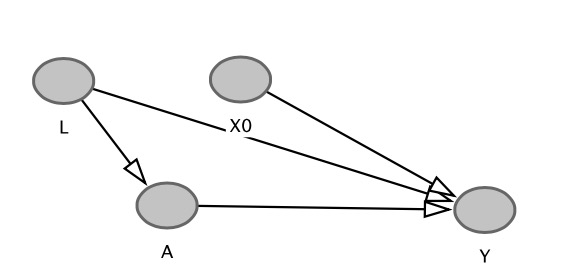


Supplementary Figure E1. Causal diagram for IPW approaches. Left: $Y$ indicates the outcome; $A$ indicates the intervention; $L$ indicates the set of identified confounding factors for $A$; $X_{0}$ indicates the set of other prognostic factors for the outcome Y. Right: $\boldsymbol{D}_{\boldsymbol{k}}$ indicates CVD status at time k (months of follow-up).


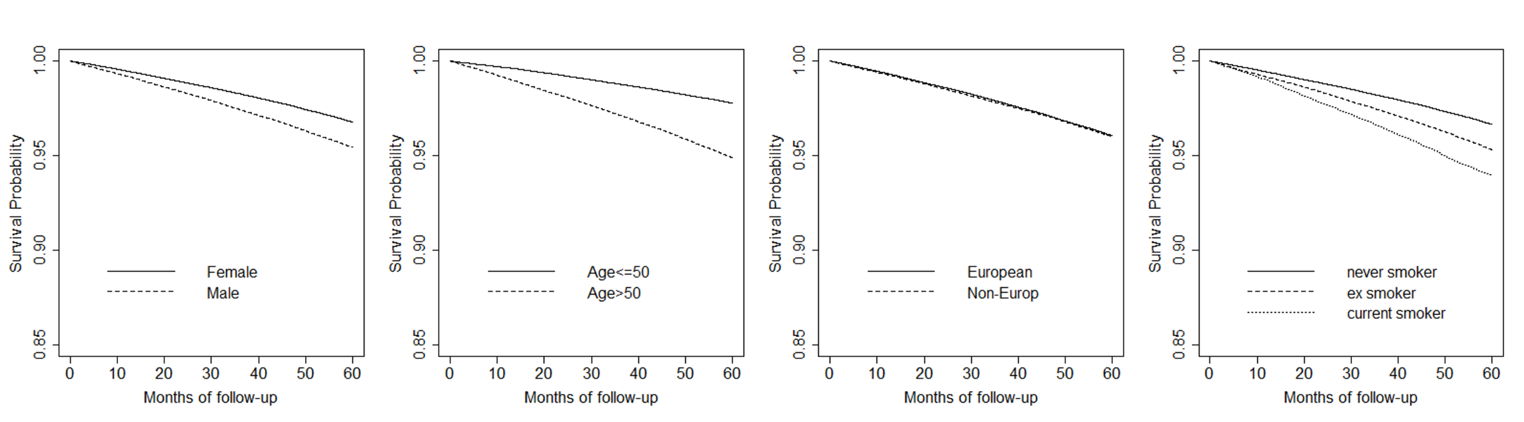


Supplementary Figure E2. Kaplan-Meier plots of survival probability in different patient groups.


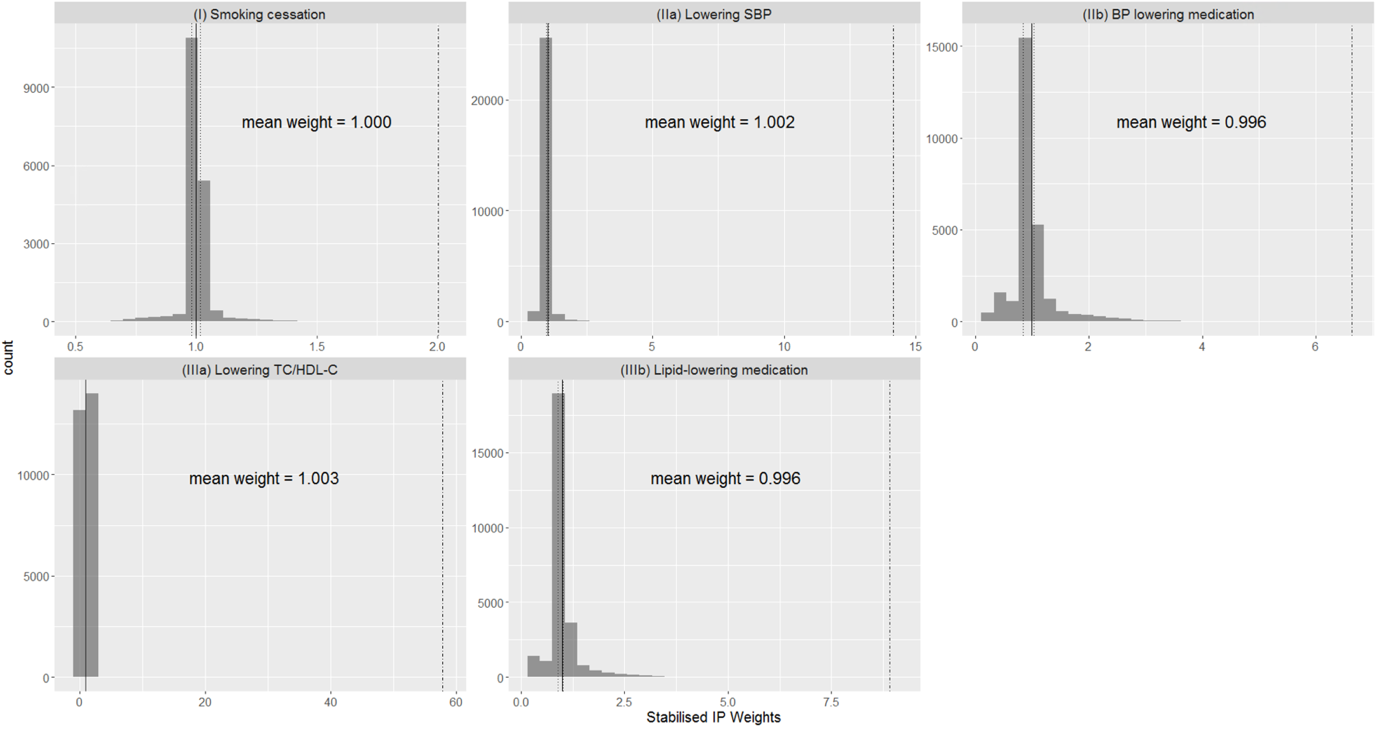


**Supplementary Figure E3.** Histograms of stabilised IP weights for different interventions. Dashed lines: lower and upper quartiles; Solid lines: mean; Dot-dash lines: maximum.


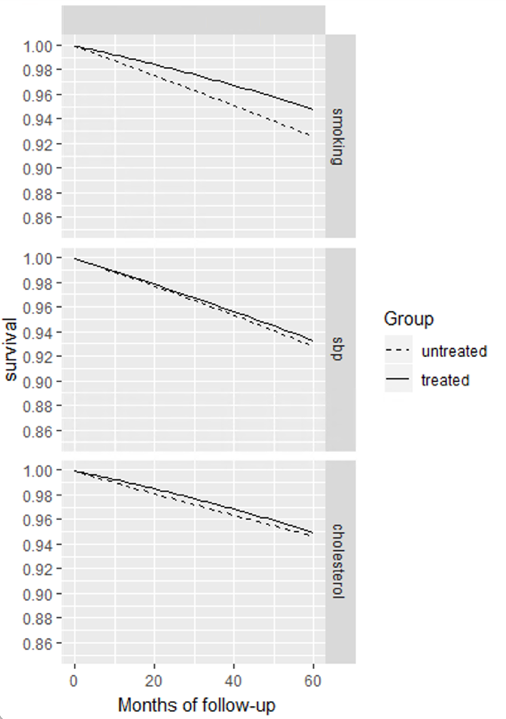

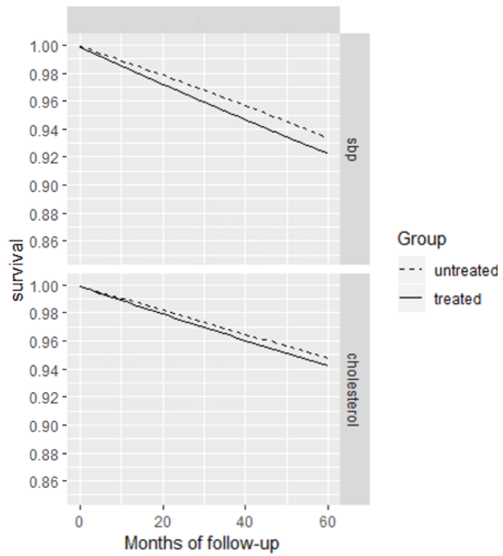


1. (B)

Supplementary Figure E4. Survival probability from an IP weighted hazards model: (A). For interventions (I) smoking cessation, (IIa) lowering SBP, (IIIa) lowering TC/HDL-C. The solid curves represent the treated group and the dotted is for untreated group. (B). For interventions (IIb) BP lowering medication and (IIIb) lipid lowering medication. The solid curves represent the treated group and the dotted is for untreated group.


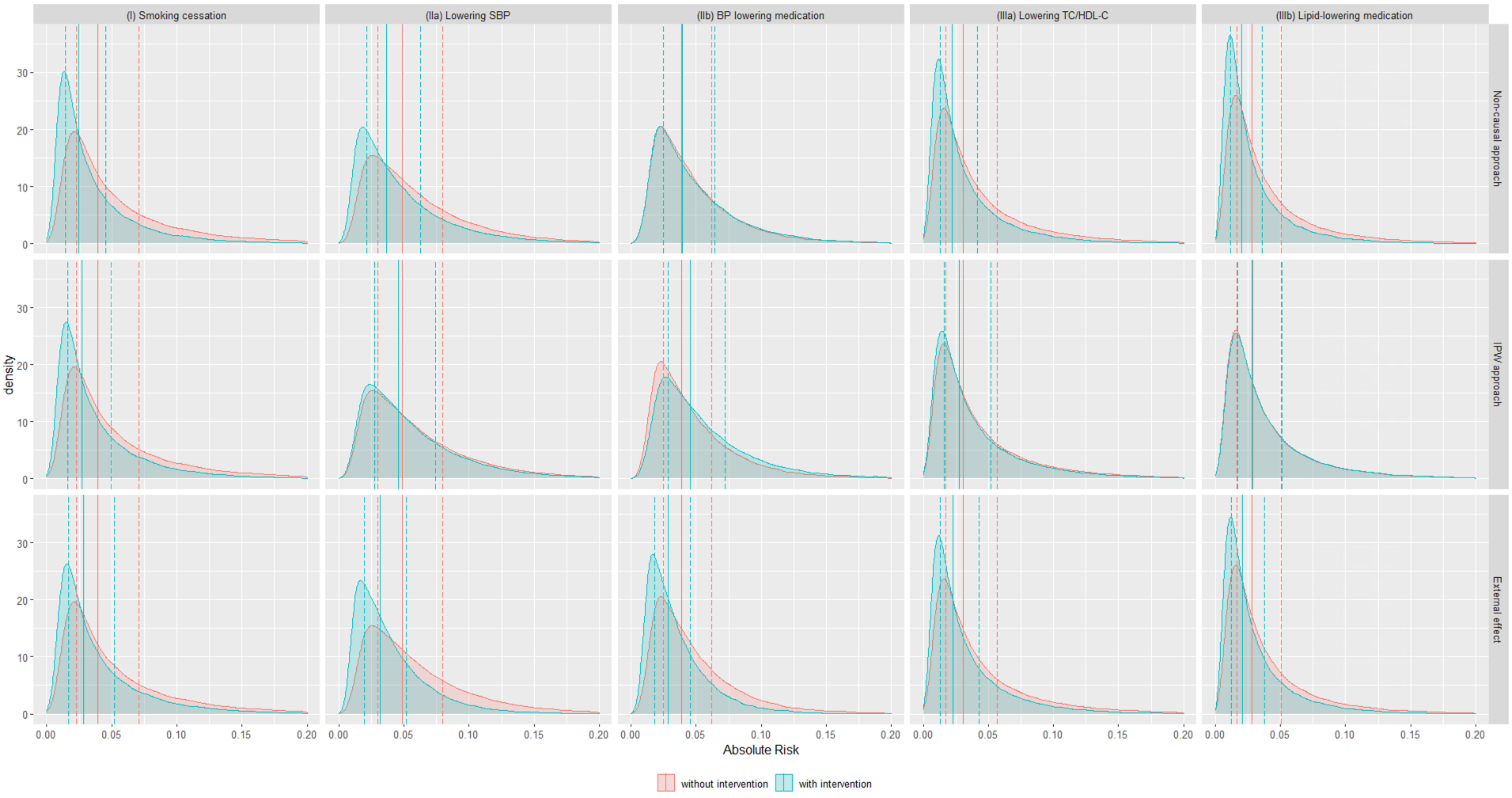


**Supplementary Fig**ure **E5.** Distribution of absolute risk estimated from different approaches in different scenarios: (I) Smoking cessation, (IIa) Lowering SBP, (IIb) BP lowering medication, (IIIa) Lowering TC/HDL-C, and (IIIb) Lipid lowering medication. Blue, with interventions; Red, without interventions; Dashed lines: lower and upper quartiles; Solid lines: median.

## Supplementary Tables

Supplementary Table E1. Baseline characteristics and outcomes of the PREDICT cohort for current smokers at baseline. The table describes all current smokers at baseline (N=66,458) as well as the groups with follow-up within 2 years from baseline (N=18,258, included in the IPW approach) and without follow-up within 2 years from baseline (N=48,200).

Data are N (%) unless indicated otherwise. NZ Dep=New Zealand Index of Socioeconomic Deprivation (a numeric value from 1 to 5, lowest to highest deprivation quintile). SBP=systolic blood pressure. TC/HDL=total cholesterol to HDL cholesterol ratio.

|  | **Total** | **With follow-up within 2 years from baseline** | **No follow-up** |
| --- | --- | --- | --- |
|  | (N=66458) | (N=18258) | (N=48200) |
| **Age(years), Mean (SD)** | 49.9(9.2) | 52.3(9.5) | 49.0(8.9) |
| **Sex** |  |  |  |
| Female | 24943(37%) | 6747(36%) | 17646(37%) |
| Male | 42065(63%) | 11909(64%) | 301567(63%) |
| **SBP, Mean mmHg (SD)** | 128.8(16.2) | 133.4(17.2) | 127.0(15.4) |
| **TC/HDL, Mean (SD)** | 4.4(1.4) | 4.6(1.5) | 4.3(1.3) |
| **Incident total CVD within 5yrs from baseline** | 15577(3%) | 4531(4%) | 11046(3%) |
| **Ethnicity** |  |  |  |
| European | 26505(40%) | 7262(39%) | 19243(40%) |
| **NZ Dep, Mean (SD)** | 3.6(1.4) | 3.7(1.4) | 3.6(1.4) |
| **Family history of premature CVD** | 7851(12%) | 2707(15%) | 5144(11%) |
| **Diabetes** | 6933(10%) | 4613(25%) | 2320(5%) |
| **Atrial fibrillation** | 338(0.5%) | 149(0.8%) | 189(0.5%) |
| **Medications at baseline** |  |  |  |
| Lipid-lowering medication | 3397(5%) | 2296(12%) | 1101(2%) |
| Antithrombotic medication | 2106(3%) | 1441(8%) | 665(1%) |
| BP-lowering medication | 3953(6%) | 2541(14%) | 1412(3%) |

Supplementary Table E2. Beta coefficients from refitted CPM and current PREDICT model. CVD risk from PREDICT model can be calculated by: $\left( \boldsymbol{1-}\text{baseline surv }^{\exp\left( \boldsymbol{sum of coefficients \times variables} \right)} \right)\boldsymbol{\times100.}$

NZDep=New Zealand Index of Socioeconomic Deprivation. SBP=systolic blood pressure. TC/HDL-C=total cholesterol to HDL cholesterol ratio. OBPLM=on blood pressure-lowering medications. OLLM=on lipid-lowering medications. OATM=on antithrombotic medications. Baseline survival (at 5 years) is the survival predicted with all predictors at their means.

|  | **PREDICT**  **(female)** | **refitted PREDICT**  **(female)** | **PREDICT**  **(male)** | **refitted PREDICT (male)** |
| --- | --- | --- | --- | --- |
| Age | 0.0756412 | 0.072087754 | 0.0675532 | 0.0676234 |
| Maori | 0.3910183 | 0.465883913 | 0.2899054 | 0.35781349 |
| Pacific | 0.2010224 | 0.314346777 | 0.1774195 | 0.31043198 |
| Indian | 0.1183427 | 0.095860408 | 0.2902049 | 0.18276439 |
| Asian | -0.28551 | -0.33859176 | -0.3975687 | -0.4127755 |
| NZDep quintile | 0.1080795 | 0.113142957 | 0.0794903 | 0.07044576 |
| Ex-smoker | 0.087476 | 0.127872115 | 0.0753246 | 0.0872162 |
| Current-smoker | 0.6226384 | 0.647034588 | 0.5058041 | 0.51977242 |
| Family history of CVD | 0.0445534 | 0.066267029 | 0.1326587 | 0.13616994 |
| Atrial fibrillation | 0.8927126 | 0.894545374 | 0.5880131 | 0.5637951 |
| Diabetes | 0.5447632 | 0.500767257 | 0.5597023 | 0.49456508 |
| SBP per 1 mmHg | 0.0136606 | 0.018439897 | 0.0163778 | 0.01897567 |
| TC/HDL-C | 0.1226753 | 0.135519996 | 0.1283758 | 0.13732456 |
| OBPLM | 0.339925 | 0.381825062 | 0.2947634 | 0.31370106 |
| OLLM | -0.0593798 | -0.06155674 | -0.0537314 | -0.0180492 |
| OATM | 0.1172496 | 0.164681863 | 0.0934141 | 0.0677013 |
| Age x Diabetes | -0.0222549 | -0.016455946 | -0.020235 | -0.0159106 |
| Age x SBP | -0.0004425 | -0.000538926 | -0.0004184 | -0.0005784 |
| OBPLM x SBP | -0.004313 | -0.007287935 | -0.0053077 | -0.0045176 |
| Baseline survival function (at 5 years) | 0.983169213 | 0.985567573 | 0.974755526 | 0.97765173 |

Supplementary Table E3. Estimated relative risks estimated by IPW Approach where data from the 1^st^ follow-up after baseline were used to estimate treatment effects.

|  | **IPW effects** |
| --- | --- |
| (I) Smoking cessation | 0.73 (0.38–1.07) |
| (IIa) Lowering SBP | 0.99 (0.70–1.29) |
| (IIb) BP lowering medication | 1.16 (0.69–1.62) |
| (IIIa) Lowering TC/HDL-C | 0.95 (0.53–1.36) |
| (IIIb) Lipid lowering medication | 1.13 (0.61–1.65) |

**Supplementary Table E4.** C-index and AIC for treatment models in estimating inverse probability weights: linear models versus models with cubic splines applied to continuous variables (age, SBP, and TC/HDL ratio).

|  | (I) Smoking cessation | (IIa) Lowering SBP | (IIb) BP lowering medication | (IIIa) Lowering TC/HDL-C | (IIIb) Lipid lowering medication |
| --- | --- | --- | --- | --- | --- |
| C-index Linear | 5.6117E-01 | 6.3615E-01 | 7.6253E-01 | 7.1474E-01 | 7.8138E-01 |
| C-index Spline | 5.7314E-01 | 6.3621E-01 | 7.6267E-01 | 7.1890E-01 | 7.8216E-01 |
| AIC Linear | 1.2661E+04 | 3.1722E+04 | 2.4595E+04 | 1.1170E+04 | 1.9804E+04 |
| AIC Spline | 1.2643E+04 | 3.1728E+04 | 2.4591E+04 | 1.1121E+04 | 1.9792E+04 |

Supplementary Table E5. Absolute risk changes (ARC): median and lower and upper quartiles (LQ, UQ) across the target population, estimated from different approaches. ARC = risk without intervention – risk under intervention.

|  | **Non-causal approach using refitted PREDICT:**  **ARC median (LQ, UQ)** | **IPW**  **ARC median (LQ, UQ)** | **Effects from external sources: ARC median (LQ, UQ)** |
| --- | --- | --- | --- |
| (I) Smoking cessation | 1.42% (0.85%, 2.56%) | 1.17% (0.69%, 2.13%) | 1.05% (0.62%, 1.92%) |
| (IIa) Lowering SBP | 0.90% (0.55%, 1.55%) | 0.34% (0.21%, 0.56%) | 1.71% (1.04%, 2.78%) |
| (IIb) BP lowering medication | -0.18% (-0.60%, 0.19%) | -0.62% (-0.99%, -0.40%) | 1.05% (0.67%, 1.67%) |
| (IIIa) Lowering TC/HDL-C | 0.78% (0.43%, 1.49%) | 0.27% (0.16%, 0.51%) | 0.76% (0.44%, 1.42%) |
| (IIIb) Lipid lowering medication | 0.77% (0.44%, 1.42%) | -0.06% (-0.10%, -0.03%) | 0.70% (0.41%, 1.26%) |

# Reference

1. Velentgas P, Dreyer NA, Nourjah P, Smith SR, Torchia MM, editors. Developing a Protocol for Observational Comparative Effectiveness Research: A User’s Guide [Internet]. Rockville (MD): Agency for Healthcare Research and Quality (US); 2013 [cited 2022 Mar 11]. (AHRQ Methods for Effective Health Care). Available from: http://www.ncbi.nlm.nih.gov/books/NBK126190/

2. Rubin DB. The design versus the analysis of observational studies for causal effects: parallels with the design of randomized trials. Stat Med. 2007 Jan 15;26(1):20–36.

3. Brookhart MA, Schneeweiss S, Rothman KJ, Glynn RJ, Avorn J, Stürmer T. Variable selection for propensity score models. Am J Epidemiol. 2006 Jun 15;163(12):1149–56.

4. Myers JA, Rassen JA, Gagne JJ, Huybrechts KF, Schneeweiss S, Rothman KJ, et al. Effects of Adjusting for Instrumental Variables on Bias and Precision of Effect Estimates. Am J Epidemiol. 2011 Dec 1;174(11):1213–22.

5. VanderWeele TJ, Shpitser I. A new criterion for confounder selection. Biometrics. 2011 Dec;67(4):1406–13.

6. Pearl J. Remarks on the method of propensity score. Stat Med. 2009 Apr 30;28(9):1415–6; author reply 1420-1423.
